# Effects of Fine Particulate Matter and Its Components on Emergency Room Visits for Pediatric Pneumonia: A Time-Stratified Case-Crossover Study

**DOI:** 10.3390/ijerph182010599

**Published:** 2021-10-10

**Authors:** Ming-Ta Tsai, Yu-Ni Ho, Charng-Yen Chiang, Po-Chun Chuang, Hsiu-Yung Pan, I-Min Chiu, Chih-Min Tsai, Fu-Jen Cheng

**Affiliations:** 1 Department of Emergency Medicine, Kaohsiung Chang Gung Memorial Hospital, No. 123, Ta-Pei Road, Niao-Sung, Kaohsiung 833, Taiwan; kabadada@gmail.com (M.-T.T.); r223054987@cgmh.org.tw (Y.-N.H.); bestmulatto@gmail.com (C.-Y.C.); bogy1102@cgmh.org.tw (P.-C.C.); gettingfat720@gmail.com (H.-Y.P.); outofray@hotmail.com (I.-M.C.); 2 College of Medicine, Chang Gung University, No. 259, Wenhua 1st Road, Guishan District, Taoyuan City 333, Taiwan; tcmnor@cgmh.org.tw; 3 Department of Pediatrics, Kaohsiung Chang Gung Memorial Hospital, No. 123, Ta-Pei Road, Niao-Sung, Kaohsiung 833, Taiwan

**Keywords:** particulate matter, particulate matter component, air pollution, pediatric, pneumonia

## Abstract

Pneumonia, one of the important causes of death in children, may be induced or aggravated by particulate matter (PM). Limited research has examined the association between PM and its constituents and pediatric pneumonia-related emergency department (ED) visits. Measurements of PM_2.5_, PM_10_, and four PM_2.5_ constituents, including elemental carbon (EC), organic carbon (OC), nitrate, and sulfate, were extracted from 2007 to 2010 from one core station and two satellite stations in Kaohsiung City, Taiwan. Furthermore, the medical records of patients under 17 years old who had visited the ED in a medical center and had a diagnosis of pneumonia were collected. We used a time-stratified, case-crossover study design to estimate the effect of PM. The single-pollutant model demonstrated interquartile range increase in PM_2.5_, PM_10_, nitrate, OC, and EC on lag 3, which increased the risk of pediatric pneumonia by 18.2% (95% confidence interval (Cl), 8.8–28.4%), 13.1% (95% CI, 5.1–21.7%), 29.7% (95% CI, 16.4–44.5%), 16.8% (95% CI, 4.6–30.4%), and 14.4% (95% Cl, 6.5–22.9%), respectively. After PM_2.5_, PM_10_, and OC were adjusted for, nitrate and EC remained significant in two-pollutant models. Subgroup analyses revealed that nitrate had a greater effect on children during the warm season (April to September, interaction *p* = 0.035). In conclusion, pediatric pneumonia ED visit was related to PM_2.5_ and its constituents. Moreover, PM_2.5_ constituents, nitrate and EC, were more closely associated with ED visits for pediatric pneumonia, and children seemed to be more susceptible to nitrate during the warm season.

## 1. Introduction

Pneumonia is one of the main infectious diseases that are responsible for childhood morbidity and death, leading to about 1.3 million mortalities among children less than 5 years old in 2010 and 2011 [1]. Pneumonia is an inflammatory condition of the lung, and previous studies have shown a relationship between air pollution and lung inflammation [2,3]. Recently, many epidemiologic studies have demonstrated that PM_2.5_ (particulate matter (PM) with an aerodynamic diameter of <2.5 μm) has a greater hazardous effect than other air pollutants [4,5]. For children, a review article concluded that PM_2.5_ was positively related to child admission due to pneumonia [6]. Furthermore, the health impact of PM_2.5_ seemed to present regional heterogeneity. Bell et al. gathered data from 202 counties in the United States and discovered the strongest relationship between PM_2.5_ and respiratory hospitalizations in the northeast region [7]. Regional variation may be explained by some community features, such as the prevalence of air conditioning [8], the percentage of elderly residents [9], and weather conditions [10]. Another possible reason for regional heterogeneity was the different PM components in different regions [11]. The PM_2.5_ constituent, nitrate, was found to have a greater influence on asthma emergency department (ED) visits [12], while sulfate was found to be more associated with daily mortality [13]. Limited studies have focused on PM components and pediatric pneumonia, and their results were inconclusive [14].

On the other hand, the health effect of PM_2.5_ seems to have seasonal variation. Lv et al. found that children were more sensitive to pneumonia due to PM_2.5_ during the warm season [15], and Ueda et al. revealed that the influence of PM_2.5_ on daily mortality was more significant during transitional seasons [13]. The combination of climate conditions and PM seemed to enhance the hazardous effect on health [16]. The seasonal effect of PM components on pediatric pneumonia is still obscure. As a result, this present study aimed to (1) assess the impacts of short-period exposure to PM_2.5_ and its components on pediatric pneumonia and (2) reveal the effect of PM constituents on pediatric pneumonia in different seasons.

## 2. Materials and Methods

### 2.1. Study Area and Population

A retrospective observational study was conducted between 1 January 2007 and 31 December 2010, in an urban tertiary academic medical center in Kaohsiung, Taiwan, with an average of 72,000 ED visits per year. Kaohsiung is largely populated by heavy industries, including the petrochemical industry and steel corporation situated in southwest Taiwan. This study was approved by the institutional review board of our hospital (no. 202001095B0C501) and was performed in accordance with the ethical standards set forth in the 1964 Declaration of Helsinki and its later amendments or comparable ethical standards. For this type of study, informed consent from the subjects was not required. The ED visit data of non-traumatic pediatric (<17 years) patients with a principal diagnosis of pneumonia (International Classification of Diseases, ninth revision: 480–486) were obtained. According to the ED records, data on demographic factors, such as age, sex, and pre-existing diseases, including cerebral palsy, epilepsy, and respiratory disease (including asthma), were also collected.

### 2.2. Pollutant and Meteorological Data

**Figure 1:**
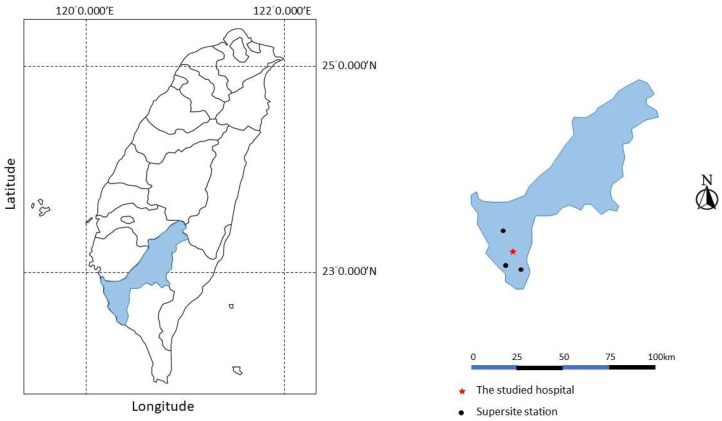
The locations of one core supersite and two satellite supersites in Kaohsiung. Note: the Taiwan map outline was adapted from https://webvectormaps.com/taiwan-map-outline-free-blank-vector-map/ (accessed on 8 October 2021), which was licensed under the Creative Commons Attribution 4.0 International License.

We measure hourly mass concentrations of PM_10_, PM_2.5_, and four major PM_2.5_ constituents, including elemental carbon (EC), organic carbon (OC), nitrate, and sulfate from the southern PM supersite during the study period. The southern PM monitoring supersite was operated by the Taiwan Environmental Protection Administration from 2006 to 2010. There was one core supersite [Fooyin (latitude 22.60° N, longitude 120.38° E)] and two satellite supersites [Chiautou (To) (22.75° N, 120.29° E) and Qianzhen (22.60° N, 120.30° E)] in Kaohsiung, as described previously [17], as shown in Figure 1. At these supersites, hourly mass concentrations of PM_2.5_ were measured with a tapered element oscillation microbalance (Rupprecht and Patashnick 1400a). Hourly concentrations of OC and EC were detected with Series 5400 Monitor, nitrate was detected with Series 8400N Particulate Nitrate Monitor, and sulfate was detected with Series 8400S Particulate Sulfate Monitor [15]. Climate conditions, such as hourly temperature and humidity, were also recorded at the core station. We measured the daily average of PM_10_, PM_2.5_, and PM_2.5_ constituents from each monitoring site, as well as from residential areas of pediatric pneumonia patients. The 24-h mean level of each pollutant and the meteorological data, including mean temperature and mean humidity from the closest monitoring supersites, were also collected for further analysis.

### 2.3. Statistics

Both time-stratified and case-crossover techniques were used to analyze the data [18]. The design is a substitute for the Poisson time series regression models for studying the heath effect of short-term exposure, such as air pollution [13,19]. In general, the case-crossover design and the time-series approach yielded almost identical results.

The method was described in our previous study [20]. Briefly, we selected referent days as the days falling on the same day of the week within the same month as the index day, in order to adjust for the influences of seasonality, long-term trends, and day of the week [21]. The day on which the pediatric patient visited ED was defined as lag 0, the day before pneumonia event was lag 1, the day before lag 1 was lag 2, and so on. The impact of each lag day was assessed separately. Conditional logistic regression was used to estimate the odds ratios (ORs) and 95% confidence intervals (CIs) of the pneumonia cases associated with PM_2.5_ and its components. Subgroup analyses, including season, temperature, sex, age, and pre-existing diseases, were also carried out to identify the most vulnerable groups.

The levels of each air pollutant and meteorological variables were entered into the models as continuous variables. The baseline model included a linear expression, including each air pollutant and meteorological confounding factors, such as temperature and humidity. Then, plotting univariate restricted cubic splines were used to evaluate the potential non-linear relationships between weather conditions and pediatric pneumonia. We used the Akaike information criterion (AIC) to examine nonlinear effects by introducing temperature and humidity separately in the model, and we compared the goodness-of-fit. SAS macro lgtphcurv9 (in SAS version 9.4) was used; it implements natural cubic spline methodology to fit a potentially nonlinear response curve in conditional logistic regression models for matched case-control studies [22]. We used both single-pollutant models and multi-pollutant models with different combinations of PM to assess the stability of the effects of PM and its components.

The ORs were calculated on the basis of per interquartile range (IQR) increments in PM_10_, PM_2.5_, nitrate, sulfate, OC, and EC exposure. Statistical significance was set at *p* < 0.05. Besides the SAS macro lgtphcurv9, other statistical analyses were performed with Statistical Product and Service Solutions version 25.0 (IBM Corp, Armonk, NY, USA).

## 3. Results

During the 4-year study period, data were recorded for 1737 ED patients with pediatric pneumonia. Demographic characteristics are presented in Table 1. In our study, the mean age was 5.1 years, and 921 (53.0%) patients were males. Pre-existing diseases were respiratory disease in 47 (0.3%) patients, cerebral palsy in 48 (0.3%), and epilepsy in 34 (0.2%). Among pediatric pneumonia cases, 867 (49.9%) patients presented during the warm season (April to September), and 801 (46.1%) patients presented on warm days (≥26.5 °C).

The meteorological factors, including the daily mean concentrations of PM and PM components in Kaohsiung during the study period, are presented in Table 2. The average concentrations of PM_2.5_ and PM_10_ were 32.7 μg/m^3^ and 50.3 μg/m^3^, respectively, during our study period. The average concentrations of nitrate, sulfate, OC, and EC were 4.4, 9.4, 8.2, and 2.1 μg/m^3^, respectively.

The Pearson’s correlation coefficients for PM_10_, PM_2.5_, each PM_2.5_ chemical constituent, and weather conditions are presented in Table 3. PM_2.5_ was significantly correlated with PM_10_ (r = 0.909; *p* < 0.001), nitrate (r = 0.793, *p* < 0.001), sulfate (r = 0.908, *p* < 0.001), and OC (r = 0.822, *p* < 0.001) and moderately correlated with EC (r = 0.669, *p* < 0.001).

**Figure 2:**
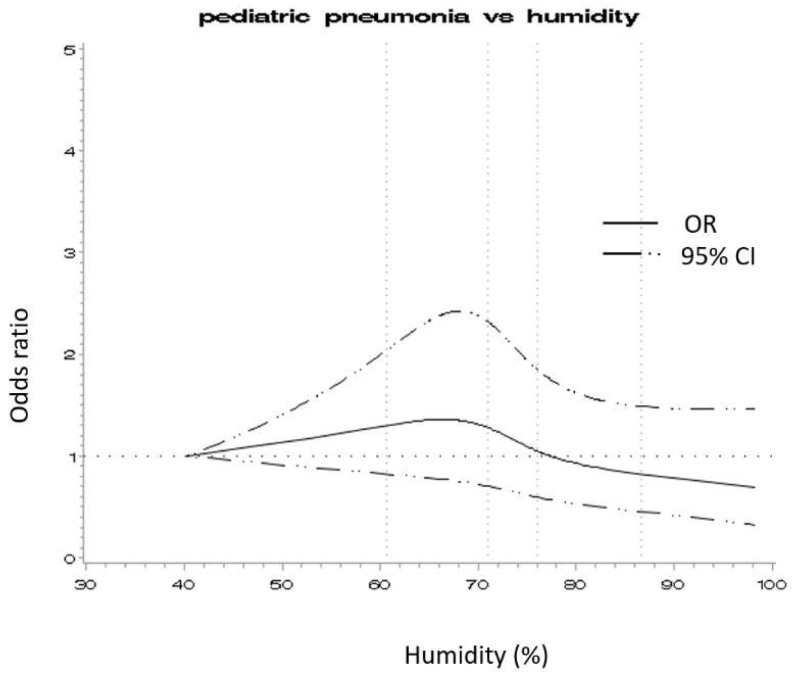
Restricted cubic spline for humidity. This figure displays a restricted cubic spline from conditional logistic regression with relative humidity as the predictor and pediatric pneumonia ED visit as the outcome. The reference exposure value was set at a relative humidity of 40%, including 4 knots.

Conditional regression was performed to evaluate the influence of PM_2.5_ and its constituents on pediatric pneumonia. When the environmental temperature was set as a continuous linear term, the AIC value for the linear model (4816.364) was better than that of the spline model (4817.46), and the curvature test (nonlinear relationship) was non-significant (*p* =0.306). As shown in Figure 2, with humidity, the spline model (AIC = 4788.325) was better than the linear model (AIC = 4793.54), and the curvature test was significant (*p* < 0.001). Therefore, the spline model was used to create five categorical variables according to knots for humidity (Figure 2) using AIC [23]. When the conditional logistic regression model was included in this categorical representation of relative humidity, the AIC value was 4788.032, which was better than the linear model.

**Figure 3:**
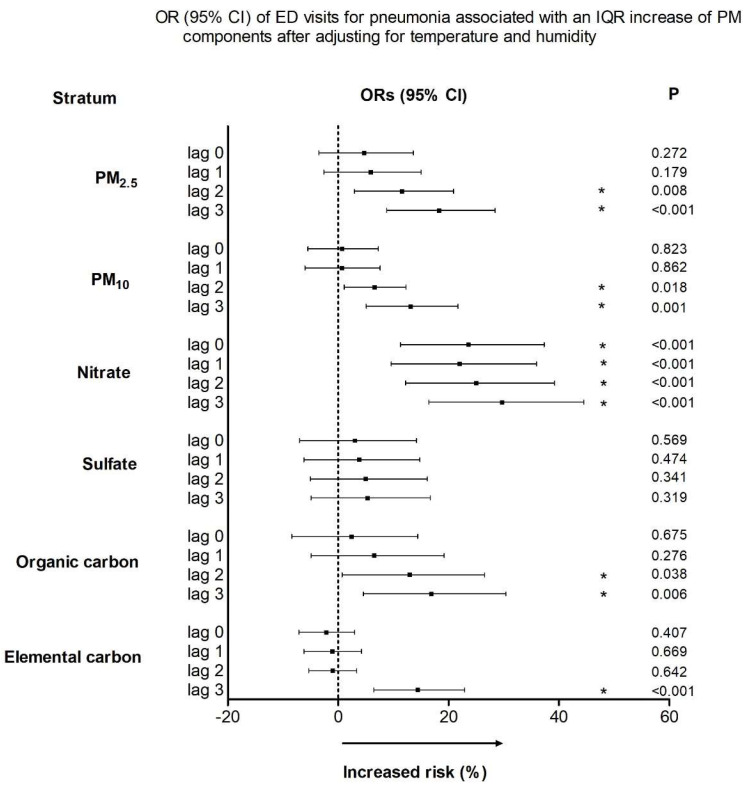
Odds ratios and 95% confidence intervals for pediatric pneumonia ED visits. The values are presented according to IQR increments in the levels of PM_2.5_ and its constituents, with adjustment for temperature and humidity. ED, emergency department; OR, odds ratio; CI, confidence interval; IQR, interquartile range; PM, particulate matter. * *p* < 0.05.

Figure 3 shows the 95% CIs, excess rates (%), and the probability that the value is different from 0 for pediatric pneumonia ED visits that are related to each IQR increase in PM_2.5_, PM_10_, and PM_2.5_ constituents after adjusting for humidity and temperature as previously described. IQR increases in PM_2.5_, PM_10_, nitrate, and OC concentrations on lag 2 were related to an increased risk of pediatric pneumonia ED visits by 11.5% (95% CI, 2.9–20.9%), 6.6% (95% CI, 1.1–12.3%), 25.0% (95% CI, 12.2–39.2%), and 12.9% (95% CI, 0.7–26.5%), respectively. IQR increases in PM_2.5_, PM_10_, nitrate, OC, and EC levels on lag 3 were related to an increased risk of pediatric pneumonia ED visits by 18.2% (95% CI, 8.8–28.4%), 13.1% (95% CI, 5.1–21.7%), 29.7% (95% CI, 16.4–44.5%), 16.8% (95% CI, 4.6–30.4%), and 14.4% (95% CI, 6.5–22.9%), respectively. On the other hand, IQR increase in sulfate level was not significantly related to pediatric ED visits.

A two-pollutant model was designed to offer insight into which sole component, independent of the effects of the other PM_2.5_ components, may enhance the risk of pediatric pneumonia ED visits (Table 4). In the multi-pollutant models, we concurrently included PM_2.5_, PM_10_, nitrate, OC, and EC on lag 3, according to the results of the single-pollutant model. As presented in Table 4, the risk of ED visits for pediatric pneumonia was significantly associated with an IQR increase in nitrate after adjusting for PM_2.5_ (OR = 1.230; 95% CI: 1.064–1.442), PM_10_ (OR = 1.250; 95% CI: 1.104–1.416), OC (OR = 1.299; 95% CI: 1.136–1.485), and EC (OR = 1.231; 95% CI: 1.095–1.384). The risk of pediatric pneumonia ED visits was significantly associated with an IQR increment in EC after adjusting for PM_2.5_ (OR = 1.091; 95% CI: 1.014–1.175), PM_10_ (OR = 1.108; 95% CI: 1.030–1.193), nitrate (OR = 1.083; 95% CI: 1.010–1.162), and OC (OR = 1.130; 95% CI: 1.029–1.242). The risk of PM_2.5_, PM_10_, and OC for pediatric pneumonia was not statistically significant after adjustment for nitrate.

**Figure 4:**
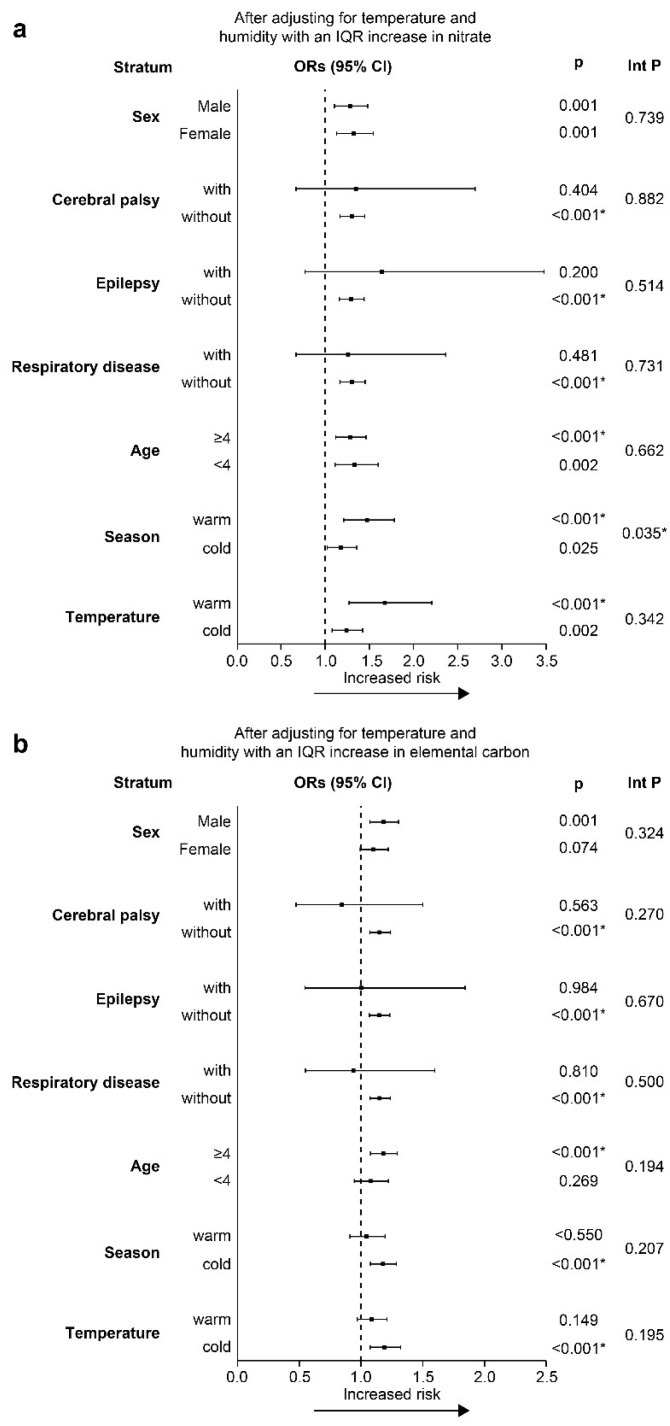
Adjusted ORs and CI of subgroup analysis for PM_2.5_ constituents on lag 3: (**a**) nitrate, (**b**) elemental carbon. The X-axis represents percentage of excess risk with 95% confidence intervals. Warm season was defined as April through September; cold season as October through March. Warm temperature, ≧26.5 °C; cold temperature, <26.5 °C. CI, confidence intervals; Int P, interaction *p* value; IQR, interquartile range; OR, odds ratio; PM, particulate matter. * *p* < 0.05.

Figure 4a,b shows the results of the stratified analysis used to evaluate the effects of nitrate and EC on pediatric pneumonia based on different age groups, environmental temperature, seasons, and underlying physical conditions on lag 3. As shown in Figure 4a, after adjusting for temperature and humidity, patients were more susceptible to nitrate during the warm season (OR = 1.469; 95% CI, 1.211–1.781; interaction *p* = 0.035). The results were not significantly different for nitrate in subgroups of different ages and underlying diseases. Figure 4b demonstrates that the effect estimates for EC were higher in male patients, in older patients (≥4 years), during the cold season, and during cold days (<26.5 °C), but the results were not statistically significant.

## 4. Discussion

Our study demonstrated that both PM_2.5_ concentration and its chemical components are related to pediatric ED visits. Among the four PM_2.5_ components that we evaluated in the study (i.e., nitrate, sulfate, OC, and EC), the estimated effects of nitrate and EC on pediatric pneumonia were more robust after adjusting for PM_2.5_, PM_10_, and OC. Furthermore, children were more susceptible to nitrate during the warm season.

Many epidemiologic studies have revealed that PM_2.5_ is related to cardiovascular diseases, respiratory diseases, and daily mortality [4,9,24], but few studies have focused on PM_2.5_ and pediatric pneumonia, and even the results of these few studies are controversial. Lv et al. and Xiao et al. demonstrated that elevated PM_2.5_ was related to pediatric hospital admission for pneumonia and ED visits for pediatric pneumonia [14,15], but some studies did not conclude on a positive correlation between ED visits for pediatric pneumonia and PM_2.5_ exposure [25,26]. The following may be explanations for this dissimilarity. First, the lag times of the studies were different. Strickland et al. collected their data up to lag 1, and Malig et al. collected their data up to lag 2. Lv et al. revealed that PM_2.5_ was associated with pediatric pneumonia admission on lag 4. Xiao et al. indicated that the 3-day moving average PM_2.5_ was related to pediatric pneumonia ED visits. The present study showed that PM_2.5_ was associated with pediatric ED visits on lag 2 and lag 3. That is to say, the studies did not find statistical significance probably due to fewer lag days traced. Second, the source and composition of PM_2.5_ may have seasonal and regional variations [11], and different constituents may cause different health impacts. Among PM_2.5_ components, chloride, EC, and OC were found to be related to cardiovascular mortality, and ammonia and potassium were related to respiratory mortality [13]. A systemic review/meta-analysis enrolled 17 studies and concluded on a positive association between hospitalization of children due to pneumonia and daily levels of PM_2.5_ [6]. Our study showed a positive relationship between PM_2.5_ and ED visits for pediatric pneumonia, which is similar to the results of previous studies.

Limited studies have focused on PM components and health hazards. Nitrate and EC were found to be related to hemorrhagic stroke, while OC and EC were found to be related to ischemic stroke [27]. EC, OC, sulfate, selenium, and silicon are associated with cardiovascular disease mortality [28]. Sanart et al. showed that EC and OC were related to ED visits for cardiovascular diseases, and PM_2.5_ was associated with ED visits for asthma/wheeze [29]. For respiratory diseases, Hwang et al. conducted a population-based study by collecting data from the National Health Insurance Research Database and concluded that PM_2.5_ was related to pediatric asthma ED visits, and asthma ED visits were significantly associated with concentrations of nitrate [12]. Furthermore, limited studies have focused on PM components and the risk of pediatric pneumonia. Xiao et al. found that PM_2.5_ and PM_2.5_ constituents (EC, OC, sulfate, and ammonium) were associated with a higher risk of ED visits for pediatric pneumonia [14]. Our study presented similar results. Our single-pollutant model revealed that PM_2.5_, EC, OC, and nitrate were related to pediatric ED visits. Furthermore, our study showed that nitrate and EC were independently associated with pediatric pneumonia, even after adjusting for PM_2.5_ and other PM_2.5_ constituents in the two-pollutant model. On the other hand, another study revealed that OC was positively related to ED visits for pediatric pneumonia, but PM_2.5_ and its components (EC, nitrate, and sulfate) were not related to pediatric ED visits [30]. This conclusion is different from our study’s and that of Xiao et al. Only patients < 4 years old were enrolled in Darrow’s study, and the difference in study groups may be the cause of the different results. Therefore, our study suggests that nitrate and EC have more hazardous effects than other PM constituents. However, the results should be interpreted with caution. Although inorganic water-soluble ionic species, such as nitrate and sulfate, and carbonaceous species, EC and OC, are the major components of PM [31], there are elemental components of PM_2.5_, such as nickel and zinc, and organic components, such as polycyclic aromatic hydrocarbons (PAHs), which were not monitored in the present study. In animal experiments, the water extracts of PM, mainly elemental components, led to an inflammatory response of the lungs and increased production of reactive oxygen species in the lungs [32]. PAHs in urban air PM may lead to persistent activation of DNA damage signaling [33]. Thus, our results demonstrate that nitrate and EC have more significant effects among PM components (EC, OC, nitrate, and sulfate) on pediatric pneumonia, but further study is needed to clarify the elemental and organic components of PM and their hazardous effect on pediatric health.

Previous studies revealed that the adverse effects of PM on human health varied with the season: Cheng et al. revealed that PM_2.5_ was positively associated with ED visits for pneumonia during the warm season [4]; Hsu et al. discovered that PM_2.5_ was related to cardiovascular hospitalization, especially in winter [34]. Few studies have focused on the seasonal effect of PM on pediatric pneumonia. A previous study demonstrated that children seemed to be more susceptible to pneumonia caused by PM_2.5_ on warm days (>23 °C); however, interaction p was not analyzed in the study [15]. Our previous study revealed that PM_2.5_ was related to pediatric pneumonia ED visits, but there was no discernible seasonal difference [35]. In the present study, a strong association was found between nitrate and pediatric pneumonia during the warm season, and there was no evident effect of seasonality between EC and pediatric pneumonia. That is to say, in different studies, the varying compositions of PM_2.5_ in different seasons and regions may lead to distinct results.

Many toxicological studies have shown the adverse health effects of PM. In animal studies, PM exposure was found to probably contribute to sinonasal inflammation, accompanied by eosinophilic inflammation, an increase in inflammatory cytokines, and eosinophil accumulation [36]. On the other hand, PM_2.5_ exposure has been explored to reduce phagocytosis of alveolar macrophages by activating proinflammatory cytokines, such as tumor necrosis factor-α, interleukin (IL)-1β, and IL-6 [37]. Human studies have also demonstrated that PM is related to systemic inflammation. Hassanvand et al. showed that there were positive associations between PM exposure and elevated white blood cells (WBCs), high-sensitivity C-reactive protein, and other pro-inflammatory cytokines, such as IL-6 and tumor necrosis factor-soluble receptor-II [38]. Pneumonia is recognized as an inflammatory condition in the lungs, and PM exposure may trigger or exacerbate lung inflammation. The current study also supports this hypothesis, and we found a positive association between PM exposure and the risk of pediatric pneumonia.

In different age groups, PM_2.5_ has been noted to have different human health effects. Lv et al. discovered that children younger than 1 year old were more susceptible to PM_2.5_ and had a higher risk of pneumonia compared to children over 1 year old [15]. On the contrary, another study revealed that children aged 1–4 years were more susceptible to PM_2.5_ and more easily had pneumonia and upper airway infection than children less than 1 year old [30]. However, interaction p was not calculated in either study. Our previous study showed that older children (aged ≥ 4 years, interaction *p* = 0.024) were more susceptible to PM_2.5_ [35]. The present study found that older children seemed more susceptible to EC, but the association did not reach statistical significance (interaction *p* = 0.194). Children of different ages may have a variation in outdoor activity and staying time in different studies. This variation may lead to distinct exposure to PM_2.5_, with different results. Additionally, the composition of PM_2.5_ may differ in seasons and regions [39], contributing to the diversity of its health effects.

Many previous epidemiologic studies have shown that the interval for the health effect of PM ranged from 0 to 3 days. Pan et al. found that PM_2.5_ concentration increases the risk of ST-segment elevation myocardial infarction on lag 0 [40]; Cheng et al. revealed a positive association between out-of-hospital cardiac arrest and PM_2.5_ concentration on lag 1 [20]. For pediatric pneumonia, Lv et al. found that PM_2.5_ was positively correlated with hospital admissions on lag 2 [15]. Xiao et al. found the joint effect of O_3_ and PM_2.5_ components (sulfate, nitrate, and ammonium) on pediatric pneumonia ED visits on lag 0–2 [14]. In the present study, we revealed a positive association between ED visits for pediatric pneumonia and PM_2.5_ on lag 2 and lag 3. There may be several possible reasons for the different time windows. First, we discovered a positive correlation between nitrate and pediatric pneumonia on lag 0 to lag 3, a positive correlation between OC and pediatric pneumonia on lag 2 to lag 3, and a positive correlation between EC and pediatric pneumonia on lag 3. Different components of PM_2.5_ seem to have different time windows for the risk of pediatric pneumonia. This is because constitutions of PM_2.5_ vary with seasons and regions, resulting in distinct time windows. Second, Hassanvand et al. revealed that exposure to PM_2.5_ was related to elevation of WBCs and IL-6; for healthy young adults, the elevation of WBC and IL-6 was on lag 1, while for the elderly (>65 years of age), WBC and IL-6 were elevated on lag 0 to lag 4. In other words, different patient groups may have different reactions and response times to PM exposure, and this may lead to diversity in study results [38].

## 5. Limitation

There are several limitations to the present study. First, our study was conducted in in a single tertiary medical center located in tropical metropolitan and industrial city. The results may, therefore, not be generalizable to other areas that have different meteorological and regional characteristics. Second, factors such as the usage of personal protective equipment, time spent outdoors, and use of air purification devices may influence people’s exposure to PM, and the observed results may vary under different conditions. Further studies should be conducted in regions with diversity, and the use of air purification devices or personal protective equipment should be factored into the analysis.

## 6. Conclusions

We discovered that PM_10_, PM_2.5_, and PM_2.5_ constituents (EC, OC, and nitrate) were positively associated with ED visits for pediatric pneumonia in Kaohsiung, Taiwan. The influences of nitrate and EC were strong after adjusting for PM_10_, PM_2.5_, and OC. Children were more susceptible to pediatric pneumonia due to nitrate during the warm season.

## Figures and Tables

**Table 1:** Demographic characteristics of patients.

All	Number = 1737	%
Demographic characteristics of patients		
Age (mean ± standard deviation)	5.1 ± 3.6	
Male sex	921	53.0
Respiratory disease	47	0.3
Cerebral palsy	48	0.3
Epilepsy	34	0.2
Warm season	867	49.9
Warm days (≥26.5 °C)	801	46.1

**Table 2:** Meteorological factors during the study period.

	Minimum	Percentiles			Maximum	Mean	IQR
		25%	50%	75%			
PM_2.5_ (µg/m^3^)	6.9	18.9	31.6	43.0	119.5	32.7	24.1
PM_10_ (µg/m^3^)	10.7	29.7	46.6	66.9	449.5	50.3	37.2
Nitrate (µg/m^3^)	0.3	1.4	3.9	6.6	20.7	4.4	5.2
Sulfate (µg/m^3^)	1.1	5.6	9.1	12.5	33.7	9.4	6.9
Organic carbon (µg/m^3^)	1.4	5.4	7.5	10.6	27.8	8.2	5.2
Elemental carbon (µg/m^3^)	0.5	1.5	2.0	2.6	16.5	2.1	1.1
Temperature (°C)	13.4	22.6	26.5	28.8	31.6	25.5	6.2
Humidity (%)	44.0	69.0	73.4	77.3	95.3	73.2	8.3

IQR, interquartile range; PM, particulate matter.

**Table 3:** Pearson’s correlation coefficients between air pollutants and weather conditions in the study period.

	PM_10_	PM_2.5_	Nitrate	Sulfate	Organic Carbon	Elemental Carbon	Temperature (°C)	Humidity (%)
**PM_10_**		0.909	0.669	0.774	0.731	0.568	−0.493	−0.410
**PM_2.5_**			0.793	0.908	0.822	0.669	−0.504	−0.406
**Nitrate**				0.680	0.833	0.643	−0.580	−0.269
**Sulfate**					0.673	0.592	−0.403	−0.359
**Organic carbon**						0.732	−0.536	−0.377
**Elemental carbon**							−0.376	−0.277
**Temperature (°C)**								0.315
**Humidity (%)**								

PM, particulate matter.

**Table 4:** OR (95% CI) of pneumonia ED visits for each interquartile range change in two-pollutant models, adjusted for temperature, relative humidity, and pollutant.

	Adjusted for PM_2.5_	Adjusted for PM_10_	Adjusted for Nitrate	Adjusted for Organic Carbon	Adjusted for Elemental Carbon
**PM_2.5_**		1.160 (1.011–1.331)	1.063 (0.951–1.189)	1.168 (1.050–1.300)	1.124 (1.024–1.233)
**PM_10_**	1.019 (0.903–1.150)		1.051 (0.966–1.144)	1.102 (1.012–1.199)	1.084 (1.002–1.173)
**Nitrate**	1.230 (1.064–1.442)	1.250 (1.104–1.416)		1.299 (1.136–1.485)	1.231 (1.095–1.384)
**Organic carbon**	1.024 (0.889–1.181)	1.081 (0.951–1.229)	0.997 (0.870–1.143)		1.028 (0.887–1.192)
**Elemental carbon**	1.091 (1.014–1.175)	1.108 (1.030–1.193)	1.083 (1.010–1.162)	1.130 (1.029–1.242)	

CI, confidence interval; ED, emergency department; OR, odds ratio; PM, particulate matter.

## Data Availability

The datasets used and analyzed during the current study are available from the corresponding author on reasonable request.

## Bullet Point:

- PM_2.5_ was significantly associated with pediatric pneumonia.
- ED visits for pediatric pneumonia were more directly related to nitrate and EC.
- Children were more susceptible to nitrate during the warm season.

## Abbreviations

AIC	Akaike information criterion
CI	confidence interval
EC	elemental carbon
ED	emergency department
IL	interleukin
IQR	interquartile range
OC	organic carbon
OR	odds ratio
PAH	polycyclic aromatic hydrocarbons
PM	particulate matter
WBC	white blood cell
